# Global longitudinal strain to predict left ventricular dysfunction in asymptomatic patients with severe mitral valve regurgitation: literature review

**DOI:** 10.1007/s12471-019-01318-8

**Published:** 2019-08-13

**Authors:** G. P. Bijvoet, A. J. Teske, S. A. J. Chamuleau, E. A. Hart, R. Jansen, J. Schaap

**Affiliations:** 1 Department of Cardiology, Maastricht University Medical Centre, Maastricht, The Netherlands; 2 Department of Cardiology, University Medical Centre Utrecht, Utrecht, The Netherlands; 3 Department of Cardiology, Amphia Hospital, Breda, The Netherlands

**Keywords:** Mitral valve insufficiency, Echocardiography, Ventricular dysfunction

## Abstract

The optimal treatment strategy for asymptomatic patients with severe mitral valve regurgitation (MR) and preserved left ventricular (LV) function is challenging. This manuscript reviews the available literature on the value of left ventricular global longitudinal strain (LV-GLS) in predicting LV dysfunction after mitral valve surgery in these patients and discusses its current place in the treatment strategy. Studies were identified from Cochrane Library, SCOPUS, PubMed and Web of Science up to February 2018. The domain used was MR. The determinant was LV-GLS; other methods of deformation imaging were excluded. The examined outcome was LV dysfunction after surgery. A total of 144 articles were retrieved, of which 11 publications met the inclusion criteria, including a total of 2415 patients. Ten studies showed a significant correlation between preoperative LV-GLS and LV dysfunction postoperatively; one study reported a negative correlation. These studies suggest that LV-GLS is a predictor of LV dysfunction after surgery in asymptomatic patients with chronic MR. Hence, incorporation of LV-GLS for clinical decision-making in these patients might be of additional value. Further research is needed to confirm the role of LV-GLS in postoperative patients, and additionally in asymptomatic MR patients during a ‘watchful waiting’ strategy.

## Introduction

The optimal treatment strategy for asymptomatic patients with severe mitral valve regurgitation (MR) and preserved left ventricular (LV) systolic function is still challenging. The risks of surgical intervention should be weighed against the benefits as regards mortality and morbidity—i.e. preventing occurrence of LV remodelling and systolic dysfunction by reducing volume overload. To determine the best treatment strategy and optimal timing of intervention, several diagnostic tools are available. Echocardiography is the principal investigation used to assess the severity and mechanism of MR and is the recommended imaging modality during follow-up [1]. LV volumes and systolic function determined by LV ejection fraction (LVEF) might be relatively late markers of myocardial dysfunction. According to the European guidelines, the use of left ventricular global longitudinal strain (LV-GLS) could be of potential interest for the detection of early subclinical LV dysfunction [1]. This article systematically reviews the available literature on the value of deformation imaging in severe MR and discusses its role in clinical decision-making in asymptomatic patients with severe MR.

### Current treatment strategies

The 2017 European guidelines for the management of valvular heart disease recommend mitral valve (MV) surgery (class I indication) in cases of severe asymptomatic MR and LV dysfunction. LV dysfunction is defined as LVEF < 60% and/or LV end-systolic diameter >45 mm [1]. Furthermore, surgery should be considered in patients with new-onset atrial fibrillation or pulmonary hypertension (class IIaB) or with left atrial dilatation or chordal rupture when a durable repair is likely and surgical risk low (class IIaC). In the absence of any of these phenomena, the optimal treatment strategy is less well-defined and two different approaches have been suggested: ‘watchful waiting’ versus early MV repair. Arguments for a watchful waiting strategy are the perioperative risks, especially when valve replacement is necessary, as well as the relatively benign natural course of asymptomatic chronic MR [2]. However, opposing observational data challenge this view on the watchful waiting strategy. Two large studies suggest that early MV surgery results in better long-term LV function and lower rates of heart failure [3, 4]. However, all available data were derived from observational studies and no randomised controlled studies have been performed to date. It should also be noted that the perioperative risks of MV repair have decreased significantly over the last few years. Yazdchi et al. [5] highlight the safety and efficacy of MV repair in an overview of all MV repairs in their centre from 1985 to 2011, a total of 5902 patients. They report an in-hospital mortality of less than 0.1% in the period 2005–2011, with hospital stay as short as 5 days, and positive results regarding postoperative MR (97% had no or mild MR at discharge and MV replacement was required in only 0.25% of patients). Considering the low perioperative risks and the notion that subclinical LV dysfunction as a result of volume overload may be present even when LVEF is still preserved, the balance may shift towards early surgery as the preferred treatment strategy for asymptomatic patients with severe MR and preserved LVEF.

### Potential role of deformation imaging in clinical decision-making

**Fig. 1:**
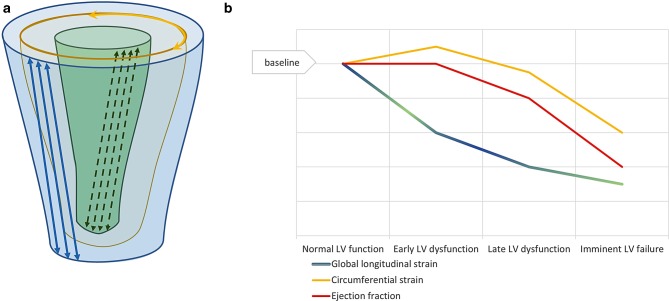
**a** Schematic drawing of left ventricular (*LV*) fibre direction, showing the longitudinal arrayed fibres in endo- and epicardial layers, and the circumferential arrayed fibres in the midwall. **b** Hypothesised change in contractile function of different fibre layers and their influence on global longitudinal strain and ejection fraction

Ideally, in an asymptomatic patient, surgery should take place when LVEF is still normal, but myocardial dysfunction is imminent. An important diagnostic tool might be the evaluation of LV-GLS. There is strong evidence that in multiple cardiac diseases the prognostic value of LV-GLS is superior to that of LVEF when it comes to predicting LV dysfunction and major adverse cardiac events [6]. A common hypothesis is that in many cardiac diseases the first change is a loss of function in longitudinal fibres in endo- and epicardial layers, while the circumferential fibres in the midwall stay relatively unaffected or even compensate by augmenting circumferential shortening. This results in a compensatory increase of circumferential shortening, counterbalancing the loss of longitudinal function and resulting in a preservation of LVEF [7]. Therefore, LV-GLS seems to be a promising parameter since this reflects early myocardial dysfunction, uncloaking dysfunction in the setting of a preserved LVEF (Fig. 1).

This manuscript reviews the available literature on the value of LV-GLS in predicting LV dysfunction after surgery in asymptomatic patients with severe MR and discusses its current place in the treatment strategy of these patients.

## Methods

We performed a literature search to collect all relevant articles to answer the following research question: ‘Is LV-GLS a predictor of LV dysfunction in asymptomatic patients with chronic MR?’

**Fig. 2:**
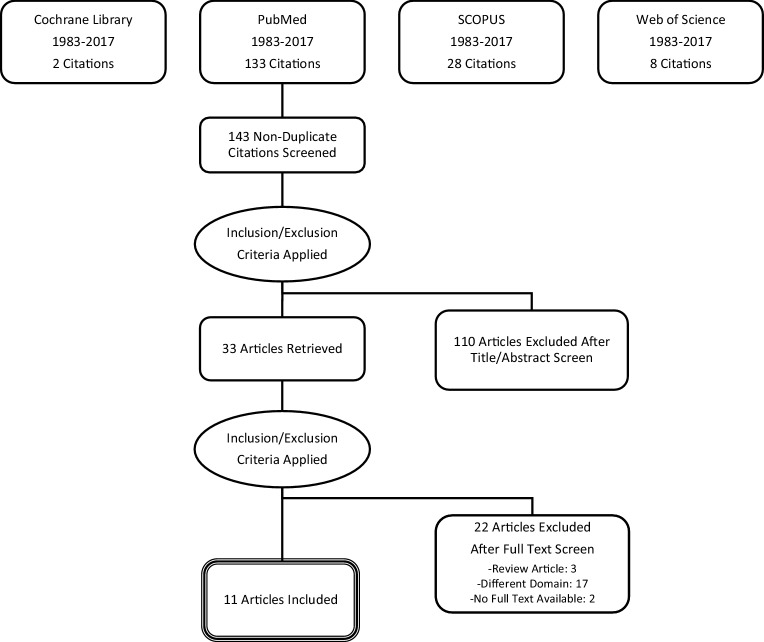
Search strategy (PRISMA flow diagram)

**Fig. 3:**
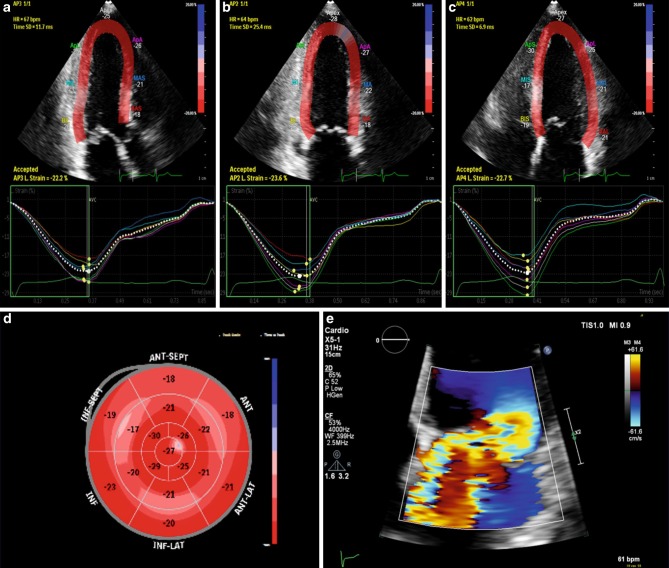
Example of a patient with severe mitral valve regurgitation (MR) and normal global longitudinal strain value. *Top* 2D apical views [*left* to *right* apical 3‑chamber view (*AP3*), apical 2‑chamber view (*AP2*), apical 4‑chamber view (*AP4*)] with strain overlay and corresponding strain curves underneath. *Bottom left* Bull’s eye pattern of regional strain values. *Bottom right* Colour Doppler image illustrating the severe MR

A search of the Cochrane Library, SCOPUS, Web of Science and PubMed was performed in February 2018 (Fig. 2), using the following keywords: ‘mitral valve insufficiency’, ‘global longitudinal strain’, ‘GLS’, ‘LV-GLS’ and ‘deformation’, with their respective synonyms and related terms. For the PubMed search this translated into the following search string: (((‘Mitral Valve Insufficiency’[Mesh]) OR ‘Mitral Valve Insufficiency’[Majr])) AND ((((global longitudinal strain[Title/Abstract]) OR GLS[Title/Abstract]) OR LV-GLS[Title/Abstract]) OR deformation[Title/Abstract]). After the exclusion of duplicates 143 studies were assessed for eligibility. Studies were included if they: (1) prospectively enrolled patients with severe MR, (2) performed standard methods to acquire LVEF as the reference for LV systolic function, (3) performed either 2D or 3D speckle tracking echocardiography (STE) or both with echocardiography and recorded LV-GLS for LV function assessment, and (4) performed credible statistical methods to analyse the prognostic value of LV-GLS determined by STE. An example of LV-GLS derived by 2D STE is shown in Fig. 3. Since the sub-domain ‘asymptomatic MR patients’ has not yet been extensively studied in this regard, studies with a mixed population of symptomatic and asymptomatic patients were also included. Studies were excluded in case of: (1) different outcome than LV dysfunction, as reflected by the endpoint (change in) LVEF or mortality, (2) different determinant than LV-GLS (e.g. regional strain, rotation or contractile reserve), (3) no full text available, (4) language other than English and (5) publication before the year 1983. After application of the in- and exclusion criteria 33 full-text articles were assessed for eligibility and 11 studies were included in this systematic review.

## Results

The results from the included studies are summarised in Tab. 1. Three studies used a short-term follow-up (<30 days). Of these only the study by Ternacle et al. [17] used the hard endpoint of mortality at 30 days. This study in 425 patients undergoing cardiac surgery for various indications (23% MV surgery) describes a significant association between abnormal LV-GLS (cut-off −18%) and 30-day postoperative death, despite a similar EuroSCORE II (odds ratio 2.4, 11.8 vs 4.9%, *p* = 0.04). The incremental value for predicting the combined end-point defined by death and the need for prolonged inotropic support was significant in all subgroup analyses, including the MR subgroup. Two studies used the endpoint of early postoperative LVEF decrease by >10%. Florescu et al. [11] studied 28 patients with asymptomatic MR and preserved ejection fraction and at 2 weeks postoperatively there was a positive correlation between LV-GLS < −18% and a postoperative decrease in LVEF by >10%. Pandis et al. [16] studied 130 patients who underwent MV repair because of severe MR and preserved ejection fraction. In contrast to all other studies, the presence of a *higher* preoperative LV-GLS (cut off −17.9%) predicted an early (3-day) postoperative reduction in LVEF of >10%. The authors attribute the conflicting finding to the patient selection (patients strictly undergoing only MV repair, thereby excluding coronary artery bypass surgery, a fixed downsized prosthetic annulus, and an extensive rheumatic process that may alter regional wall motion and deformation characteristics). They state that the higher baseline LV-GLS signifies a maladaptive preload-related change that is associated with substantial loss of LVEF (>10%) immediately after surgery. Two other important factors, not highlighted by the authors, could be:

1. The postoperative LVEF measurements were done only 3 days after surgery, whereas other studies used a longer follow-up period.
2. All LV-GLS data were collected retrospectively using TomTec software applied to echocardiographic data from different vendors from different referring hospitals. As such, critics might consider the data to be less robust.

**Table 1:** Summary of the results from the included studies

Study	*n*	Population characteristics					Outcome	Follow-up	Results		Vendor
		Age (years)	Male	Asympt	Mean LVEF	Surgery [MV repair]			Cut-off LV-GLS	Effect (95% CI)	
Alashi 2016 [8]	448	61 ± 12	69%	100%	62 ± 3%	100% [92%]	Mortality	8 ± 2 years	−20.7%	HR 1.17^b^ (1.08–1.27)	Siemens Syngo VVI
Cho 2016 [9]	43	51 ± 14	58%	100%	64 ± 6%	100% [79%]	↓ LVEF or ↑ LVEDD	3 months	−20.5%	OR 2.44^b^ (1.259–4.729)	GE Vingmed EchoPAC
Donal 2012 [10]	77	63 ± 1	67%	36%	67 ± 7%	100% [94%]	LVEF <50%	6 months	−18%	*R* = −0.42, *p* = 0.011^b^ (n/a)	GE Vivid EchoPAC
Florescu 2012 [11]	28	59 ± 13	64%	100%	64%	100% [100%]	↓ LVEF >10%	14 days	−18%	*R*^2^ = 0.70, *p* < 0.001^b^ (n/a)	GE Vivid 7 EchoPAC
Lancellotti 2008 [12]	71	61 ± 14	55%	n/a	68 ± 6%	42% [32%]	LVEF <50%	13 ± 8 months	X-increase <1.9%	*R*^2^ = 0.74, *p* = 0.001^b^ (n/a)	GE Vivid 7 EchoPAC
Magne 2012 [13]	135	60 ± 14	56%	100%	69 ± 6%	32% [22%]	Event-free survival^a^	23 ± 19 months	−20%	HR 1.14^b^ (1.04–1.26)	GE Vivid 7/9—EchoPAC
Mascle 2012 [14]	88	63 ± 13	67%	32%	66 ± 7%	100% [82%]	LVEF <50%	6 ± 1 months	−18%	OR 4.2^b^ (1.4–13)	GE Vingmed EchoPAC
Mentias 2016 [15]	737	58 ± 13	68%	100%	62%	65% [60%]	Mortality	8 ± 3 years	−21.7%	HR 1.60^b^ (1.47–1.73)	Siemens Syngo VVI
Pandis 2014 [16]	130	57 ± 14	65%	n/a	63 ± 11%	100% [100%]	↓ LVEF >10%	3 days	−17.9%	OR 0.80^c^ (0.73–0.88)	‘various’ TomTec
Ternacle 2013 [17]	425	67 ± 13	69%	n/a	51 ± 13%	23% [11%]	Mortality	30 days	−18%	OR 2.4, *p* = 0.04^b^ (n/a)	GE Vingmed EchoPAC
Witkowski 2013 [18]	233	61 ± 12	61%	35%	66 ± 9%	100% [100%]	LVEF <50%	34 ± 20 months	−19.9%	OR 23.16^b^ (6.53–82.10)	GE Vingmed EchoPAC

*n* sample size, *Asympt* asymptomatic, *LVEF* left ventricular ejection fraction, *MV* mitral valve, *LV-GLS* left ventricular global longitudinal strain, *CI* confidence interval, *X-increase* increase with exercise, *OR* odds ratio, *HR* hazard ratio

^a^ Free from cardiovascular death, MV surgery (indication symptoms or left ventricular dysfunction) and heart failure hospitalisation

^b^ Significant positive effect

^c^ Significant negative effect

Eight studies used mid- to long-term endpoints, with a follow-up duration of 3 months to 8 years. Two studies used the hard endpoint of mortality [8, 15], both in a large and strictly asymptomatic population. The study by Mentias et al. [15] was an observational study in 737 asymptomatic patients with primary severe MR, a non-dilated left ventricle and a preserved ejection fraction. Different echocardiographic parameters were determined at baseline (between 2000 and 2011), including LV-GLS. During follow-up (8 ± 3 years) 65% of patients underwent surgery; indications were pulmonary hypertension after exercise, left atrial dilatation, or flail leaflet. Results showed that a LV-GLS value below the median (−21.7%) was independently associated with mortality [hazard ratio (HR) 1.60, 95% confidence interval (CI) 1.47–1.73] and provided additive prognostic utility to previously known predictors. The study by Alashi et al. [8] described the prognostic value of LV-GLS as well as brain natriuretic peptide (BNP) and right ventricular systolic pressure (RVSP) in a surgical patient group. They included 448 asymptomatic MR patients who all underwent MV surgery between 2005 and 2008 (92% MV repair). During a follow-up period of 7.7 ± 2 years, mortality was 9%. They report that an abnormal LV-GLS (cut-off −20.7%) was independently associated with worse long-term survival (HR 1.17, 95% CI 1.08–1.27, *p* < 0.01). Furthermore, the study showed that addition of LV-GLS and BNP to a clinical model provided incremental prognostic utility [χ^2^ for long-term mortality (increase from 31–47 to 61; *p* < 0.001)]. In a subsequent letter to the editor Mentias and Desai point out that a higher baseline RVSP did not independently predict postoperative LV dysfunction and as such suggests that an abnormal LV-GLS and BNP predict LV dysfunction even before pulmonary hypertension occurs [19]. Lastly, this study showed that the mitral effective regurgitant orifice area, currently a key parameter in grading severity of MR, was not associated with mortality [HR: 1.05(0.21–5.2); *p* = 0.8]. The study by Magne et al. [13] used a combined endpoint of ‘event-free survival’ [i.e. freedom from CV-related death, MV surgery (because of symptoms or LV dysfunction) and HF hospitalisation] to show that patients with a normal LV-GLS (cut-off value −20%) had significantly better 2‑year event-free survival (73% vs 44%, *p* = 0.0004) and that LV-GLS was an independent predictor of event-free survival in multivariate analysis (HR = 1.14, 95% CI 1.04–1.26, *p* = 0.007).

The other five studies focusing on long-term outcome [9, 10, 12, 14, 18] used the endpoint LVEF decrease to <50% at different time intervals from surgery. All these studies show a positive correlation between an impaired LV-GLS and postoperative LV dysfunction in asymptomatic patients with severe MR and preserved ejection fraction. However, most these studies had relatively small sample sizes. All of the included studies used the imaging modality 2D STE to obtain LV-GLS. The study of Lancellotti et al. [12] was the only one to use LV-GLS during exercise instead of rest LV-GLS as the determinant and found that a limited LV longitudinal contractile recruitment during exercise (increase of <1.9%) predicts postoperative LV dysfunction. The study by Witkowski et al. [18] showed a very large predictive value of a decreased LV-GLS (cut-off −19.9%) as an independent predictor of postoperative LV systolic dysfunction. An odds ratio of 23.16 (95% CI 6.53–82.10) was shown after multivariate analyses including LVEF ≤ 60%, atrial fibrillation, and preoperative presence of symptoms.

It is important to realise that most of the presented studies were performed in patients undergoing surgery (8 out of 11). Consequently, many included patients were either symptomatic or had an LVEF < 60%, in addition to other IIa indications for surgery. All the included studies report a mean LVEF > 60%, albeit with a wide standard deviation. For example, the study of Witkowski et al. [18] reports that 21% of patients had an LVEF < 60%. Thus, extrapolation of these results to asymptomatic MR patients with preserved ejection fraction remains difficult. Also, in some studies it is unclear whether underlying coronary artery disease and revascularisation were potential confounders.

## Discussion

The abovementioned studies suggest that in asymptomatic patients with severe MR and preserved LVEF an impaired LV-GLS is a predictor of LV dysfunction after MV surgery. An impaired LV-GLS seems to correlate not only with a decrease in LVEF shortly after surgery (<30 days), but several large studies also suggest a correlation with mortality at long-term follow-up. As such, LV-GLS could be an important parameter in clinical decision-making in this population. However, all data were collected retrospectively and need to be validated in prospective studies.

Moreover, there are several practical difficulties to be addressed before LV-GLS can be implemented in daily practice. One practical issue concerns the cut-off value of LV-GLS in patients with severe MR. In accordance with the LVEF, LV-GLS measurements are load-dependent and as such normal strain values will be higher in patients with MR than in the general population. Therefore, the reference values obtained in a healthy population do not apply for these patients. There is still no consensus on optimal cut-off values. The studies included in this review reported a median value ranging between −17.9 and −21.7%. In the absence of a definite cut-off value, a relative change in LV-GLS from baseline could potentially be a more interesting parameter, especially during ‘watchful waiting/active surveillance’. In oncology, for example, chemotherapy-related cardiac dysfunction is defined as an LV-GLS with >15% relative reduction from baseline with preservation of LVEF [20]. Perhaps this definition of cardiac dysfunction could be extrapolated to this population, but clearly more research is needed. Only one included study reported ∆‑LV-GLS [12], and these authors measured the relative change in LV-GLS during exercise, which is different than our suggested approach.

Another issue of debate regarding LV-GLS has been the intervendor differences. A joint standardisation Task Force involving professional societies and industry was initiated to reduce intervendor variability of LV-GLS. Subsequent to this initiative in 2015 there has been a significant improvement in intervendor concordance, and intervendor reproducibility is even higher than for traditional echocardiographic parameters (e.g. LVEF and peak Doppler velocities) [21–23]. All data in the included studies were collected before 2015; hence intervendor difference could be a bias. However most studies used the same vendor (Tab. 1).

Several questions remain: Firstly, does a decreased LV-GLS predict the development of symptoms during follow-up in a watchful waiting strategy, and can a temporal relation be defined? Secondly, is a decreased LV-GLS associated with the development of important haemodynamic burden secondary to the regurgitation during follow-up in a watchful waiting strategy? Lastly, does a decreased LV-GLS predict an imminent decline in ejection fraction? Since the available studies are almost exclusively performed in the perioperative phase, there are limited data on the role of LV-GLS as a parameter in understanding the natural course of the disease. Nor are there any data on the change in LV-GLS over time during a watchful waiting strategy, or how these changes in LV-GLS reflect outcome. All these questions emphasise the need for further research in prospective studies.

Outside the scope of this article, but still important to consider, are other diagnostic tools in clinical decision-making in an asymptomatic patient with severe MR. Cardiopulmonary exercise testing can be useful to uncover subclinical decline in functional capacity. Exercise echocardiography can reveal a dynamic change in MR severity and allows assessment of the effect on pulmonary pressures. Another helpful tool might be the use of biomarkers, BNP in particular. A low plasma BNP has a high negative predictive value and may be helpful in the follow-up of asymptomatic patients [24]. However, the prognostic implications of increased BNP levels are less unequivocal. Specificity is relatively low and BNP values are influenced by several factors such as renal function, age and hypertension. Multiple observational studies have shown that BNP levels during exercise provide incremental prognostic value [25, 26]. Also, patchy fibrosis as determined by MRI (with late gadolinium enhancement) may be predictive. Myocardial strain can be MRI-derived (myocardial tagging or feature tracking) as an alternative to STE. Lastly, deformation imaging of the left atrium (LA strain and strain rate) might play a future role in predicting LA remodelling secondary to severe MR, useful for determining the optimal timing of surgery [27].

## Conclusion

In this era focussing more and more on preventive healthcare, early detection of subclinical LV dysfunction is an important goal and deformation imaging might play a key role in optimising the timing of MV intervention to prevent LV dysfunction after surgery. The presented studies suggest that LV-GLS is an independent predictor of LV dysfunction in asymptomatic patients with chronic MR undergoing surgery. As such, incorporation of LV-GLS in clinical decision-making in asymptomatic MR patients could have prognostic implications. However, current studies are all retrospective studies and mostly performed in the surgical population. As such, prospective studies are needed that focus on the role of LV-GLS as a guiding parameter during a watchful waiting strategy in asymptomatic patients with chronic MR.
